# Significance of Tuft Cells Expressing Hematopoietic Prostaglandin D Synthase During the Pathogenesis of Eosinophilic Chronic Rhinosinusitis

**DOI:** 10.3390/biomedicines14071534

**Published:** 2026-07-08

**Authors:** Kenta Hosomi, Mitsuaki Ishida, Kensuke Nakanishi, Akinori Sasaki, Takaki Inui, Tetsuya Terada, Shin-ichi Haginomori, Ko Fujimori, Yoshinobu Hirose

**Affiliations:** 1 Department of Pathobiochemistry, Faculty of Pharmacy, Osaka Medical and Pharmaceutical University, 4-20-1 Nasahara, Takatsuki City 569-1094, Osaka, Japan; ompu72123058@s.ompu.ac.jp (K.H.); ko.fujimori@ompu.ac.jp (K.F.); 2 Department of Pathology, Faculty of Medicine, Osaka Medical and Pharmaceutical University, 2-7 Daigaku-Machi, Takatsuki City 569-8686, Osaka, Japan; kensuke.nakanishi@ompu.ac.jp (K.N.); akinori.sasaki@ompu.ac.jp (A.S.); yoshinobu.hirose@ompu.ac.jp (Y.H.); 3 Division of Pathology, Osaka Medical and Pharmaceutical University Hospital, 2-7 Daigaku-Machi, Takatsuki City 569-8686, Osaka, Japan; 4 Department of General and Gastroenterological Surgery, Faculty of Medicine, Osaka Medical and Pharmaceutical University, 2-7 Daigaku-Machi, Takatsuki City 569-8686, Osaka, Japan; 5 Department of Otolaryngology and Head and Neck Surgery, Faculty of Medicine, Osaka Medical and Pharmaceutical University, 2-7 Daigaku-Machi, Takatsuki City 569-8686, Osaka, Japan; takaki.inui@ompu.ac.jp (T.I.); tetsuya.terada@ompu.ac.jp (T.T.); hagi@ompu.ac.jp (S.-i.H.)

**Keywords:** eosinophilic chronic rhinosinusitis, chronic rhinosinusitis with nasal polyps, tuft cells, prostaglandin D_2_

## Abstract

**Background/Objective:** Eosinophilic chronic rhinosinusitis (ECRS) is a subtype of chronic rhinosinusitis with nasal polyps that is characterized by abundant eosinophilic infiltration within nasal polyps. Tuft cells are epithelial chemosensory cells that are present in the normal respiratory tract and activate group-2 innate lymphoid cells through their secretion of interleukin-25 and prostaglandin (PG) D_2_. ECRS is also characterized by the activation of group-2 innate lymphoid cells; however, the involvement of tuft cell-derived PGD_2_ in this process remains unclear. **Methods:** We selected consecutive patients with and without ECRS who underwent biopsy or surgical resection. Dual immunohistochemical analyses were performed to determine the presence of tuft cells producing PGD_2_, using POU class 2 transcription factor (POU2F3), a specific tuft cell marker, and hematopoietic prostaglandin D synthase (H-PGDS). **Results:** The cohort included 52 and 14 patients with and without ECRS, respectively. The number of total POU2F3-positive tuft cells (POU2F3^+^/H-PGDS^+^ and POU2F3^+^/H-PGDS^−^) was significantly higher in the ECRS group vs. the non-ECRS one (*p* < 0.0001). Moreover, the ratio of POU2F3-positive tuft cells expressing H-PGDS [POU2F3^+^/H-PGDS^+^/(POU2F3^+^/H-PGDS^+^ + POU2F3^+^/H-PGDS^−^)] was also significantly higher in the ECRS group vs. the non-ECRS one (*p* = 0.0084). **Conclusions:** These results suggest that tuft cells present in the context of ECRS may serve as a potential source of PGD_2_, thus involving to the amplification of type 2 inflammation.

## 1. Introduction

Eosinophilic chronic rhinosinusitis (ECRS) is a refractory subtype of chronic rhinosinusitis with nasal polyps (CRSwNP) that is characterized by abundant eosinophilic infiltration within nasal polyps, clinical dysosmia, long-term nasal obstruction, and nasal discharge [1,2]. In the United States and Europe, patients with chronic rhinosinusitis (CRS) are classified as either CRSwNP or CRS without nasal polyps (CRSsNP) [3]. Notably, most patients with CRSwNP in these regions have rich eosinophilic infiltration in their nasal polyps and show a high recurrence rate of these polyps after surgical removal, whereas <50% of patients with CRSwNP in East Asia experience eosinophilic-dominant inflammatory changes [4,5]. These findings suggest that the clinicopathological characteristics and underlying mechanisms of CRS vary depending on geographic and ethnic backgrounds. In Japan, the term ECRS was introduced in 2001 to refer to the subgroup of patients with CRSwNP who have abundant eosinophilic infiltration within their nasal polyps [4]. The diagnostic criteria for ECRS include which side(s) is/are affected (unilateral or bilateral) by nasal polyps, the main site involved (e.g., the ethmoid sinus), and peripheral blood eosinophilic counts [6]. Final ECRS diagnoses are made through microscopic examinations of affected lesions, wherein the mean number of eosinophils present in three high-power fields (400× magnification) must be ≥70 [6].

Tuft cells are solitary epithelial chemosensory cells that are present on the normal luminal mucosa of the respiratory and gastrointestinal tracts [7]. They characteristically express taste receptors and act as luminal sensors that detect environmental stimuli [7,8,9,10]. POU class 2 homeobox 3 (POU2F3), also known as Oct 11 or Skn 1, is the master regulator of human tuft cells [11]. Immunohistochemical analyses conducted using this marker have revealed the presence and distribution of tuft cells in various human tissues and pathological conditions [12,13,14,15]. It has been well recognized that tuft cells in the respiratory and gastrointestinal mucosa play important roles in bacterial and parasitic immune reactions, tissue repair, as well as the activation of group-2 innate lymphoid cells (ILC2) through the secretion of various physiologically active substances such as IL-25 and prostaglandin (PG) D_2_ [7,8,9,10].

The pathogenesis of ECRS has been thoroughly investigated [1,2]. Previous studies have revealed that the activation of type 2 inflammatory responses may be induced by bacterial toxins (e.g., *Staphylococcus aureus*), as well as various pathogens. Briefly, the production of interleukin (IL)-25 and IL-33 by nasal epithelial cells may lead to the increased proliferation of ILC2s, in turn leading to increased production of IL-4, IL-5, and IL-13 [1,2]. These inflammatory cascades promote eosinophilic infiltration and mucus production in the nasal and paranasal sinuses [1,2]. The immunological characteristics of ECRS are shared with the function of tuft cells [7,8,9,10]. Accordingly, recent studies have demonstrated that tuft cells are enriched in CRSwNP, and serve as a major source of IL-25 that contributes to the persistence of type 2 inflammation [16,17,18]. PGD_2_, produced by hematopoietic prostaglandin D synthase (H-PGDS), is a lipid mediator that has been implicated in a number of allergic and eosinophilic diseases such as asthma, allergic rhinosinusitis, and atopic dermatitis [19]. Moreover, it has been well recognized that tuft cells produce PGD_2_ [7,8,9,10]. However, whether the tuft cells present during ECRS produce PGD_2_ has not yet been analyzed. Therefore, this study aimed to investigate the expression of H-PGDS in the tuft cells of ECRS tissues, as well as discuss the pathogenic roles of these cells in ECRS.

## 2. Materials and Methods

### 2.1. Patient Selection

We selected consecutive patients with nasal polyps who underwent biopsy or resection at the Department of Otolaryngology and Head and Neck Surgery at Osaka Medical and Pharmaceutical University Hospital (Osaka, Japan) between January 2022 and February 2025. The criteria proposed by the Japanese Epidemiological Survey of Refractory Eosinophilic Chronic Rhinosinusitis Study was used to establish ECRS diagnoses via the following aspects: affected side: bilateral, 3 points; with nasal polyps, 2 points; computed tomography changes, ethmoid/maxillary ≥1, 2 points; and peripheral blood eosinophil ratio (%): <2–≤5%, 4 points; 5–≤10%, 8 points; and <10%, 10 points [6]. Total scores of ≥11 points were taken to indicate a high probability of ECRS, whereas definitive diagnoses were made through histological examinations of tissue samples from the patients’ nasal polyps or paranasal sinuses. Under this approach, ECRS was diagnosed if ≥70 eosinophils were identified per high-power microscope field (400× magnification) in three distinct fields [6]. Patients with nasal polyps containing low eosinophilic infiltration were also analyzed for comparison, as the non-ECRS group.

This retrospective, single-institution study was conducted in accordance with the principles of the Declaration of Helsinki, and its protocol was approved by the Institutional Review Board of Osaka Medical and Pharmaceutical University (approval no.: 2023-198; approval date: 26 March 2025). All patient data were anonymized. The institutional review board waived the requirement for written informed patient consent because of the retrospective study design, as all of the medical records and archived samples analyzed were done so with no risk to the participants. This study did not include any minors. Information regarding the study, such as the inclusion criteria and opportunity to opt out, was provided through the institution’s website (https://www.ompu.ac.jp/u-deps/path/img/file34.pdf) (accessed on 16 May 2026).

### 2.2. Histopathological Analysis

Biopsied or surgically resected specimens were fixed in 10% buffered neutral formalin, sectioned, and stained with hematoxylin and eosin. Two researchers (K.H. and M.I.) independently evaluated the histopathological features of the slides. Eosinophil counts were performed for all specimens. If ≥70 eosinophils were identified per high-power field, across three fields of one sample, the corresponding patient was diagnosed with ECRS following consideration of the other relevant clinical criteria [6].

### 2.3. Immunohistochemical Analyses

Dual immunohistochemical staining was performed using a Leica Bond-MAX autostainer (Leica Biosystems GmbH, Nußloch, Germany), according to the manufacturer’s instructions and the protocol described in our previous study [13]. The BOND Polymer Refine Detection Kit (DS9800; Leica Biosystems GmbH) and BOND Polymer Refine Red Detection Kit (DS9390; Leica Biosystems GmbH) were used to perform dual immunohistochemical staining. A rabbit monoclonal antibody against POU2F3 (E5N2D; Cell Signaling Technology, Danvers, MA, USA; diluted 1:200) and a rabbit polyclonal antibody against H-PGDS (the same one used in two of our prior related studies [13,20]; diluted 1:4000) were used. First, anti-POU2F3 primary antibody was applied to the sections after heat antigen retrieval and horseradish peroxidase-labelled secondary antibody was applied. Sections were incubated in 3-3’-diaminobenzidine; then, nuclei were labelled brown. Subsequently, heat treatment was performed for deactivation of antibody and enzyme. Then, anti-H-PGDS primary antibody was applied to the sections. Alkaline phosphatase-labelled secondary antibody was applied. Sections were incubated in Fast Red, then, nuclei and cytoplasm were labelled red. Skin squamous cells were used as positive controls for POU2F3 [15], and placental trophoblasts were used for H-PGDS [21]. Negative controls were prepared without primary antibodies. Nuclear, and nuclear and cytoplasmic staining were defined as positive immunoreactivity for POU2F3 [13,14,15] and H-PGDS [13,20,21], respectively. Mast cells present in the stroma of the nasal mucosa served as internal positive controls for both POU2F3 and H-PGDS [22,23]. Two authors (K.H. and M.I.) independently evaluated all immunohistochemical features.

POU2F3^+^/H-PGDS^+^ (nuclei stained black and cytoplasm stained red), POU2F3^+^/H-PGDS^−^ (only nuclei stained brown), and POU2F3^−^/H-PGDS^+^ cells (both nuclei and cytoplasm stained red) were counted separately in the five high-power fields with the most positive cells present (400× magnification) within the respiratory epithelium of each specimen, for both the ECRS and non-ECRS groups. The average number of positive cells were recorded.

### 2.4. Statistical Analyses

Correlations between pairs of groups containing categorical variables were analyzed using a chi-squared test. If data distribution did not follow a normal distribution, a Mann–Whitney *U* test was applied. These statistical analyses were performed using Statcel 5 (OMS Ltd., Tokyo, Japan). Statistical significance was set at *p* < 0.05.

## 3. Results

### 3.1. Patient Characteristics

Table 1 summarizes the clinicopathological features of the study cohort. We divided the cohort into ECRS and non-ECRS groups (52 and 14 patients, respectively). This study included 27 (51.9%) women and 25 (48.1%) men in the ECRS group, and six (42.9%) women and eight (57.1%) men in the non-ECRS one. The median age at the time of nasal biopsy or surgery was 52 (range 27–79) years in the ECRS group, and 51 (range 33–80) years in the non-ECRS one. Thirty-six patients (69.2%) in the ECRS group had a history of asthma. None of the non-ECRS group had a history of asthma, allergic rhinitis, and elevated blood eosinophilic ratio. No systemic corticosteroid therapy was performed in all patients.

### 3.2. Histopathological Characteristics

**Figure 1:**
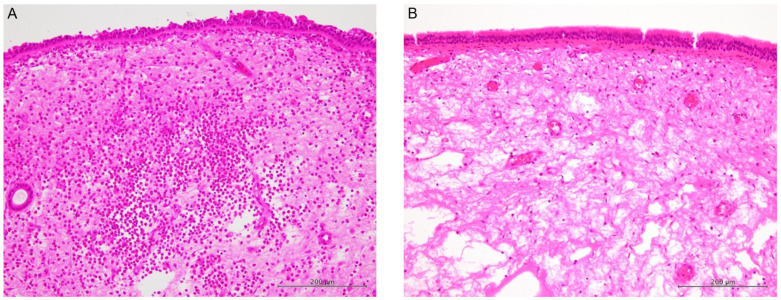
Histopathological features. (**A**) Eosinophilic chronic rhinosinusitis. Abundant eosinophilic infiltration is observed in the stroma of the nasal polyps (hematoxylin and eosin staining, ×200). (**B**) Non-eosinophilic chronic rhinosinusitis. Edematous change is observed in the stroma. Some inflammatory cells are noted, but very few eosinophils are present (hematoxylin and eosin staining, ×200).

Abundant eosinophils (>70 per high-power field) were observed in the nasal polyps of all patients in the ECRS group (Figure 1A). Conversely, edematous changes were noted in the stroma of the nasal polyps, but few eosinophils were identified (typically <5 per high-power field) in the non-ECRS group (Figure 1B).

### 3.3. Immunohistochemical Characteristics

**Figure 2:**
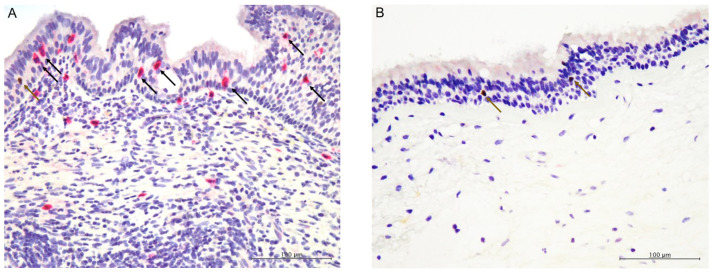
Dual immunohistochemical features for POU2F3 and H-PGDS. (**A**) Eosinophilic chronic rhinosinusitis. POU2F3^+^/H-PGDS^+^ (nuclei stained black and cytoplasm stained red) tuft cells (black arrows) are observed within the respiratory epithelium. Some POU2F3^+^/H-PGDS^−^ (only nuclei stained brown) tuft cells (brown arrow) are also noted. POU2F3^+^/H-PGDS^+^ mast cells are also present in the stroma (×400). (**B**) Non-eosinophilic chronic rhinosinusitis. Some POU2F3^+^/H-PGDS^−^ (only nuclei stained brown) tuft cells (brown arrow) are also noted (×400). H-PGDS, hematopoietic-type prostaglandin D synthase; POU2F3, POU domain class 2 transcription factor 3.

**Figure 3:**
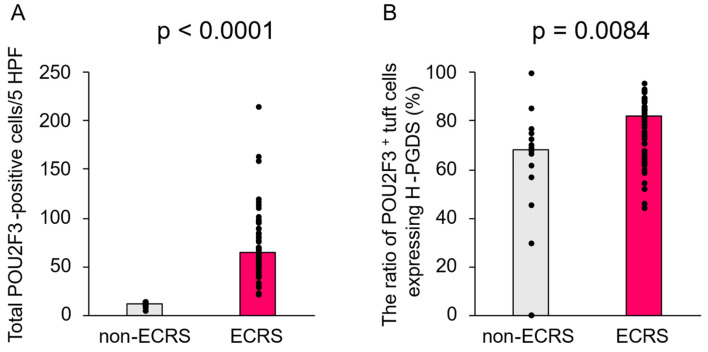
(**A**) The number of total POU2F3^+^ tuft cells per five high-power fields is significantly higher in the ECRS group vs. the non-ECRS one. (**B**) The ratio of POU2F3-positive tuft cells expressing H-PGDS is significantly higher in the ECRS group. ECRS, eosinophilic chronic rhinosinusitis; H-PGDS, hematopoietic-type prostaglandin D synthase; HPF, high-power field; POU2F3, POU domain class 2 transcription factor 3.

POU2F3-positive tuft cells were present in all of the patient samples from both the ECRS and non-ECRS groups (Figure 2A,B). The total number of POU2F3-positive tuft cells (POU2F3^+^/H-PGDS^+^ and POU2F3^+^/H-PGDS^−^) was significantly higher in the ECRS group vs. the non-ECRS one (*p* < 0.0001), as the median numbers were 65 (range 21–215) and 11 (range 4–14) cells, respectively (Figure 3A).

H-PGDS-expressing tuft cells were observed in both the ECRS and non-ECRS groups (Figure 2A,B). The ratio of POU2F3-positive tuft cells expressing H-PGDS (POU2F3^+^/H-PGDS^+^/[POU2F3^+^/H-PGDS^+^ and POU2F3^+^/H-PGDS^−^]) was also significantly higher in the ECRS group vs. the non-ECRS one (*p* = 0.0084), as the median ratios were 82.0% (range 44.2–95.5%) and 67.9% (range 0–100%), respectively, in these groups (Figure 3B).

**Figure 4:**
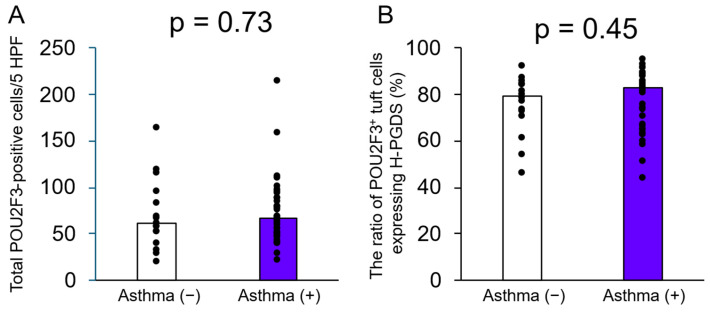
(**A**) The number of total POU2F3^+^ tuft cells per five high-power fields is not significantly different between patients with or without asthma in the ECRS group. (**B**) The ratio of POU2F3-positive tuft cells expressing H-PGDS is not significantly different between patients with or without asthma in the ECRS group. ECRS, eosinophilic chronic rhinosinusitis; H-PGDS, hematopoietic-type prostaglandin D synthase; HPF, high-power field; POU2F3, POU domain class 2 transcription factor 3.

The total number of POU2F3-positive tuft cells was not significantly different between patients with or without asthma in the ECRS group (*p* = 0.73) (Figure 4A), and the ratio of POU2F3-positive tuft cells expressing H-PGDS was not significantly different between patients with or without asthma in the ECRS group (*p* = 0.45) (Figure 4B).

**Figure 5:**
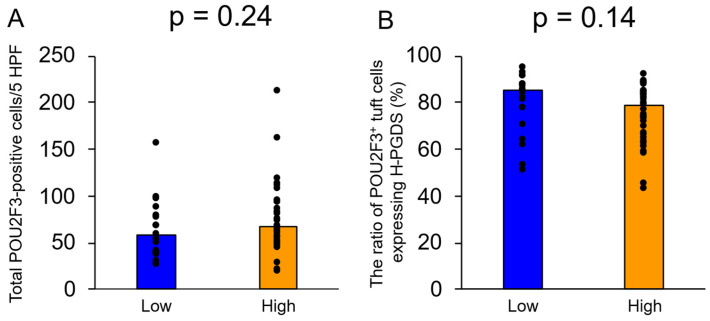
(**A**) The number of total POU2F3^+^ tuft cells per five high-power fields is not significantly different between patients with low and high eosinophilic infiltration in the nasal polyps in the ECRS group. (**B**) The ratio of POU2F3-positive tuft cells expressing H-PGDS is not significantly different with low or high eosinophilic infiltration in the nasal polyps in the ECRS group. ECRS, eosinophilic chronic rhinosinusitis; H-PGDS, hematopoietic-type prostaglandin D synthase; HPF, high-power field; POU2F3, POU domain class 2 transcription factor 3.

Moreover, the total number of POU2F3-positive tuft cells was not significantly different between patients with low and high eosinophilic infiltration in the nasal polyps in the ECRS group (*p* = 0.24) (Figure 5A). If ≥200 eosinophils (the median number of eosinophils present in the nasal polyps of the present cohort) were identified per high-power field in the nasal polyps, they were classified as the high group and if <200 eosinophils were identified, they were classified as the low group. The ratio of POU2F3-positive tuft cells expressing H-PGDS was not significantly different between patients with low and high eosinophilic infiltration in the nasal polyps in the ECRS group (*p* = 0.14) (Figure 5B).

## 4. Discussion

To our knowledge, this study is the first to demonstrate that the number of POU2F3-positive tuft cells increased significantly in ECRS, alongside the ratio of tuft cells expressing H-PGDS. Although previous articles have already demonstrated that tuft cells are enriched in ECRS [16,17,18] and tuft cells produce PGD_2_ in the gastrointestinal tract [7,8,9,10], this study clearly showed abundant presence of tuft cells expressing H-PGDS in ECRS. These findings suggest that tuft cells—particularly those that express H-PGDS—may play important roles in the pathogenesis of ECRS, particularly concerning the activation of type 2 inflammation via ILC2s, although this is a preliminary exploratory study.

It is well known that the activation of type 2 inflammation is a hallmark of ECRS, although various types of cytokines and chemokines are involved [1,2,17,18]. Type 2 inflammatory responses can be elicited by various allergens or parasitic helminths. Accumulating evidence has shown that ILC2s are the major source of type 2 cytokines such as IL-5 and IL-13, which play pivotal roles in type 2 inflammation [24]. Various pathogens can trigger the release of IL-25 and IL-33 in patients with ECRS, which activate ILC2 and Th2 cells to produce type 2 cytokines (e.g., IL-5) in the nasal mucosal environment. This, in turn, can lead to the accumulation of excess eosinophils [17,18,25,26,27,28]. IL-25 appears to be an epithelial-derived cytokine [26]; however, Patel et al. clearly demonstrated that tuft cells (alternately referred to as solitary chemosensory cells) in the respiratory epithelia represent the primary epithelial source of IL-25 in ECRS lesions [17,18]. Those studies also noted significantly higher numbers of tuft cells in ECRS lesions—which agree with our present results—as well as increased numbers of ILC2s in patients with ECRS [17,18]. Moreover, a feed-forward loop is known to exist between tuft cells and ILC2s in the small intestine, because IL-13 secreted by ILC2s enhances tuft cell differentiation in the intestinal mucosal crypt [25,26]. A similar interaction between ILC2s and tuft cells has also been demonstrated in ECRS [17].

Tuft cells are being increasingly recognized as key regulators of mucosal immunity in barrier tissues, including the gastrointestinal and respiratory tracts [10]. They play pivotal roles in immune responses to parasitic and bacterial infections, by secreting various physiologically active substances such as IL-25, thymic stromal lymphopoietin (TSLP), PGD_2_, and acetylcholine [7,10]. In the intestine, parasitic substances trigger IL-25 production by tuft cells, leading to the activation of ILC2s. Activated ILC2s then secrete IL-13, which produces type 2 inflammation alongside the proliferation of tuft and goblet cells to clear the causative parasites [10]. In the respiratory tract, tuft cells have been implicated in the regulation of eosinophilic inflammation, particularly through their interactions with ILC2 and Th2 cells [29]. Tuft cells are significantly more abundant in the respiratory epithelia of patients with ECRS vs. those without, as was demonstrated in the present study, as well as previous ones [16,17,18]. The physiologically active substances secreted by tuft cells in the respiratory epithelium, including IL-25 and acetylcholine, have been hypothesized to contribute to the development of ECRS [16,17,18]. TSLP is an epithelial-derived cytokine that represents a critical mediator of the type 2 immune response, promoting Th2-mediated conditions such as asthma and atopic dermatitis, as well as the activation of ILC2s [30]. The mRNA level of TSLP has been shown to increase in ECRS [31]. Intestinal tuft cells are known to produce TSLP, which leads to the activation of ILC2s [26,32], although it remains unclear whether tuft cells in the respiratory epithelium produce TSLP. Further analyses are therefore warranted to fully clarify the roles of tuft cells in the respiratory epithelium, as well as in the pathogenesis of ECRS.

**Figure 6:**
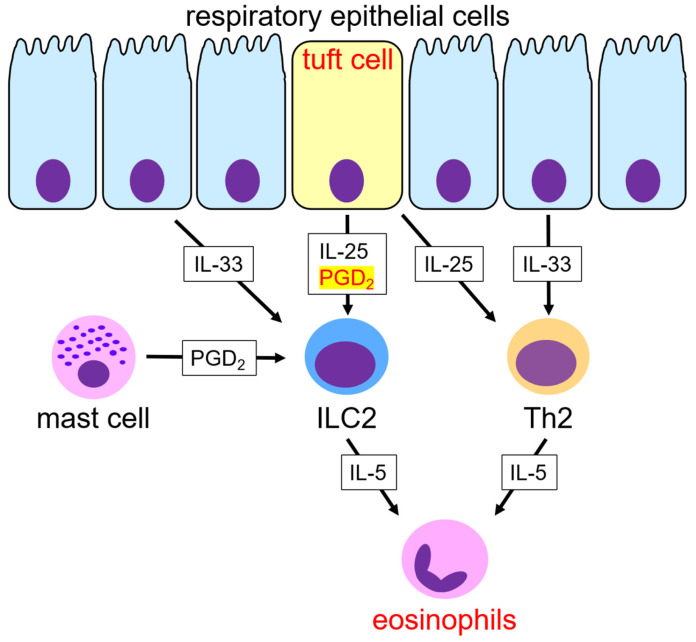
Proposed mechanism of the pathogenesis of eosinophilic chronic rhinosinusitis based on the literature [1,2,17,18,36] and the findings of the present study. IL-25 and PGD_2_, secreted by tuft cells, activate ILC2s. Mast cells are also a source of PGD_2_. ILC2, and Th2 cells, activated by IL-25 and IL-33, secrete IL-5, leading to the accumulation of eosinophils. PGD_2_, prostaglandin D2; ILC2s, group-2 innate lymphoid cells.

PGD_2_ is a lipid mediator that promotes the recruitment and activation of eosinophils, Th2 cells, and ILC2s via the chemoattractant receptor-homologous molecule expressed on the Th2 cell (CRTH2) receptor, which amplifies type 2 immune responses [33]. Elevated PGD_2_ levels and enhanced CRTH2 signaling have been reported in patients with asthma and allergic diseases, with pharmacological inhibition of these pathways demonstrating high efficacy as a treatment [34,35]. PGD_2_ has been shown to play a role in ECRS development, with one of the major sources of PGD_2_ in ECRS being mast cells [36]. Immunohistochemical analysis of H-PGDS represents a particularly useful method for detecting PGD_2_ production [13,20,21]. In this study, H-PGDS-expressing mast cells were present in the lesions of patients with ECRS. Moreover, 82.0% of the tuft cells in the ECRS group expressed H-PGDS, and these cells were significantly more abundant in the ECRS group. Tuft cells, alongside mast cells, therefore represent a potential source of PGD_2_ under conditions of ECRS, involving the activation of Th2 and ILC2 cells via CRTH2 receptor signaling, although the main source of PGD_2_ in ECRS (tuft cells or mast cells) remains unresolved. Additional studies are needed to clarify the detailed roles and sources of PGD_2_ in ECRS. Tuft cells produce various physiologically active substances in ECRS (e.g., IL-25 and PGD_2_), and play pivotal roles in the pathogenesis of ECRS through the activation of type 2 inflammation (Figure 6). Not all tuft cells expressed H-PGDS in ECRS in the present study, and this finding corresponded to the results of tuft cells in Warthin tumors [13]. Although this might be related to functional subsets or differentiation stages of tuft cells [7], additional study is needed to clarify the functions of tuft cells. Moreover, in the present ECRS cohort, the number of tuft cells and the ratio of tuft cells expressing H-PGDS were not significantly different between patients with or without asthma and low or high eosinophilic infiltration in the nasal polyps.

This study was subject to several limitations worth noting. First, being based on immunohistochemical analyses, it did not provide direct evidence of PGD_2_ production or functional activity. However, immunohistochemical analysis for H-PGDS has been proven to represent a useful method for detecting PGD_2_ production, and this dual immunohistochemical study demonstrated that POU2F3-positive tuft cells expressed H-PGDS. Second, the upstream and downstream signaling pathways and receptor-specific effects of PGD_2_ were not examined. Therefore, the possibility that pathogenetic roles of tuft cells and PGD_2_ in ECRS is speculative cannot be ruled out. Further analyses are warranted to clarify the molecular signaling pathways related to tuft cells in ECRS. Third, we used the nasal polyps containing low eosinophilic infiltration as a comparison, non-ECRS group in the present study. These tissues were not completely normal nasal mucosa because edematous change were noted and a few inflammatory cells were included. We used these tissues because of the difficulty of obtaining completely normal nasal mucosa and for comparing the mechanisms of eosinophilic infiltration in ECRS, since ECRS and non-ECRS groups form polypoid lesions. The possibility that the use of these tissues as a non-ECRS group influenced the presence of tuft cells and/or expression of H-PGDS cannot be completely ruled out. Fourth, this study included 14 patients in the non-ECRS group. Thus, the possibility of statistical bias due to an imbalance of the number of patients of groups cannot be completely ruled out. Moreover, H-PGDS expression ratio of non-ECRS group ranged 0–100%. Although this may be due to the low number of POU2F3-positive tuft cells in non-ECRS group, the presence of heterogeneity of tuft cells in non-ECRS group cannot be denied. Finally, POU2F3 was used as a human tuft cell marker in this study. Immunohistochemical analysis using other tuft cell markers, such as doublecortin-like kinase 1 (DCLK1), was not performed. DCLK-1 is specific to mouse tuft cells, but not human ones [7]; and POU2F3 is a master regulator of tuft cell identity [7]. Therefore, POU2F3 was chosen as a human tuft cell marker for the purposes of this study.

## 5. Conclusions

This study clearly demonstrated that the number of tuft cells expressing H-PGDS was significantly higher in patients with ECRS vs. those without, although this is a preliminary exploratory study for evaluating the roles of tuft cells and PGD_2_ in ECRS. Such respiratory epithelium tuft cells may represent a potential source of PGD_2_ in ECRS. Tuft cells may play important roles in the pathogenesis of ECRS (Figure 4), because they can secrete both PGD_2_ and IL-25—which represent the main mediators of the type 2 immune responses that are activated in ECRS. This makes tuft cells an important potential therapeutic target for treating ECRS. Further studies are therefore warranted to fully clarify the pathogenic roles of tuft cells in ECRS.

## Figures and Tables

**Table 1:** Clinicopathological and immunohistochemical features of ECRS and non-ECRS groups.

Variables		ECRS (N = 52)	Non-ECRS (N = 14)	*p*-Value
Median age, years	(Range)	52 (27–79)	51 (33–80)	0.32
Sex				
	Male	25 (48.1%)	8 (57.1%)	0.55
	Female	27 (51.9%)	6 (42.9%)	
History of asthma		36 (69.2%)	0	<0.0001
POU2F3-positive tuft cells in five high-power fields (cells)				
	Median (range)	65 (21–215)	11 (4–14)	<0.0001
Ratio of POU2F3-positive cells expressing H-PGDS (%)				
	Median (range)	82.0 (44.2–95.5)	67.9 (0–100)	0.0084

ECRS, eosinophilic chronic rhinosinusitis; H-PGDS, hematopoietic prostaglandin D synthase; POU2F3, POU domain class 2 transcription factor 3.

## Data Availability

All data generated and analysed in this study are included in this article.

## Abbreviations

CRSsNP	chronic rhinosinusitis without nasal polyps
CRSwNP	chronic rhinosinusitis with nasal polyps
CRTH2	chemoattractant receptor-homologous molecule expressed on Th2 cells
ECRS	eosinophilic chronic rhinosinusitis
H-PGDS	hematopoietic prostaglandin D synthase
ILC2	group-2 innate lymphoid cells
PGD_2_	prostaglandin D_2_
POU2F3	POU domain class 2 transcription factor 3
TSLP	thymic stromal lymphopoietin
